# A novel approach for assessing bias during team-based clinical decision-making

**DOI:** 10.3389/fpubh.2023.1014773

**Published:** 2023-05-09

**Authors:** Natalie Pool, Megan Hebdon, Esther de Groot, Ryan Yee, Kathryn Herrera-Theut, Erika Yee, Larry A. Allen, Ayesha Hasan, JoAnn Lindenfeld, Elizabeth Calhoun, Molly Carnes, Nancy K. Sweitzer, Khadijah Breathett

**Affiliations:** ^1^School of Nursing, University of Northern Colorado, Greeley, CO, United States; ^2^School of Nursing, University of Texas-Austin, Austin, TX, United States; ^3^Department of General Practice, University of Utrecht, Utrecht, Netherlands; ^4^Division of Cardiovascular Medicine Research Department, Indiana University, Indianapolis, IN, United States; ^5^College of Medicine, Department of Medicine and Pediatrics, University of Michigan, Ann Arbor, MI, United States; ^6^College of Medicine, University of Arizona, Tucson, AZ, United States; ^7^Division of Cardiovascular Medicine, University of Colorado, Denver, CO, United States; ^8^Division of Cardiovascular Medicine, Ohio State University, Columbus, OH, United States; ^9^Division of Cardiovascular Medicine, Vanderbilt University, Nashville, TN, United States; ^10^Department of Population Health, University of Kansas, Kansas City, KS, United States; ^11^Department of Medicine, University of Wisconsin, Madison, WI, United States; ^12^Division of Cardiovascular Medicine, Washington University in St. Louis, St. Louis, MO, United States; ^13^Division of Cardiovascular Medicine, Indiana University, Indianapolis, IN, United States

**Keywords:** methodology, qualitative descriptive analysis, bias, mixed-methods analyses, decision-making, group decision

## Abstract

Many clinical processes include multidisciplinary group decision-making, yet few methods exist to evaluate the presence of implicit bias during this collective process. Implicit bias negatively impacts the equitable delivery of evidence-based interventions and ultimately patient outcomes. Since implicit bias can be difficult to assess, novel approaches are required to detect and analyze this elusive phenomenon. In this paper, we describe how the de Groot Critically Reflective Diagnoses Protocol (DCRDP) can be used as a data analysis tool to evaluate group dynamics as an essential foundation for exploring how interactions can bias collective clinical decision-making. The DCRDP includes 6 distinct criteria: challenging groupthink, critical opinion sharing, research utilization, openness to mistakes, asking and giving feedback, and experimentation. Based on the strength and frequency of codes in the form of exemplar quotes, each criterion was given a numerical score of 1–4 with 1 representing teams that are interactive, reflective, higher functioning, and more equitable. When applied as a coding scheme to transcripts of recorded decision-making meetings, the DCRDP was revealed as a practical tool for examining group decision-making bias. It can be adapted to a variety of clinical, educational, and other professional settings as an impetus for recognizing the presence of team-based bias, engaging in reflexivity, informing the design and testing of implementation strategies, and monitoring long-term outcomes to promote more equitable decision-making processes in healthcare.

## Introduction

Within healthcare, multidisciplinary teams make numerous consensus-based clinical decisions with life and death consequences for patients. For example, during the management of advanced heart failure, multidisciplinary teams make critical decisions about surgical and non- surgical treatments (1). Stereotype-based implicit and explicit bias exhibited by individual providers is negatively associated with the allocation of appropriate advanced heart failure therapies among women and African American patients (1, 2). Because many of the contraindications for approving therapies are subjective and linked to ambiguous social factors, there is a risk of introducing bias during this high-stake collective decision-making process.

Implicit biases among healthcare providers occur at the same level as the general public and include associations outside conscious awareness that may lead to negative evaluations of a person on the basis of characteristics such as race or gender (3). However, implicit biases among clinical teams are challenging to measure due to a plethora of interpersonal dynamics, power hierarchies, and structural factors (4). Thus, the motivation for conducting additional research was to enhance our detection and understanding of implicit bias during the planning and implementation of evidence-based heart failure interventions.

It is critical to examine group dynamics for bias prior to implementation of evidence-based interventions as a counterstrategy for the harmful effects of racism, sexism, hierarchy, and other negative social constructs that contribute to health inequity. Current frameworks for understanding the implementation or pre-implementation process among multidisciplinary healthcare teams largely rely on theoretical concepts about organizational culture that are not easily operationalized in real-world settings. For example, Normalization Process Theory (NPT) can assist in identifying structural and contextual factors inhibiting the adoption of new approaches or technologies in healthcare, but it relies on four constructs that are sometimes difficult for evaluators to measure (5). In the parent study described below, NPT served as a framework for conceptualizing how complex social processes influenced clinical thinking, behavior, and practices at the group level during team decision-making. In addition, NPT provided deeper insight into how implicit bias was embedded and normalized into group-based discussions about patients during team meetings. When combined with additional metrics, this insight may stimulate the design and testing of novel interventions to better address bias among clinical teams.

Although the negative impact of provider bias on patient outcomes is known, there is a need to better understand how interpersonal interactions within a healthcare team allow biases to influence critical decision-making processes and potentially hinder the provision of equitable care. Thus, a qualitative descriptive study was conducted as part of a larger mixed-methods investigation that sought to evaluate group dynamics as an essential foundation for exploring how types of interaction can bias collective clinical decision-making. In this paper, we provide researchers and clinicians with a practical application of the DCRDP protocol to assess team interactions for implicit bias, structural racism, and inequities that influence collective clinical decision-making.

## Overview of the de Groot Critically Reflective Diagnoses Protocol

The de Groot Critically Reflective Diagnoses Protocol (DCRDP) is a mixed-methods research tool for evaluating verbal interactions among a team. The DCRDP was originally developed as a means of analyzing knowledge sharing, decision-making, and critical dialogues within professional communities (6). These behaviors can be challenging to assess and describe, yet more functional teams perform better and exhibit continual learning (6). The underlying premise of our application of the tool in this study was that more functional healthcare teams would exhibit less bias toward the patients they collaboratively cared for, although this had not explicitly been tested using the DCRDP until now. Reliability and validity of the DCRDP was previously established in studies examining reflective dialogue among veterinary and healthcare professionals (6, 7).

**Figure 1:**
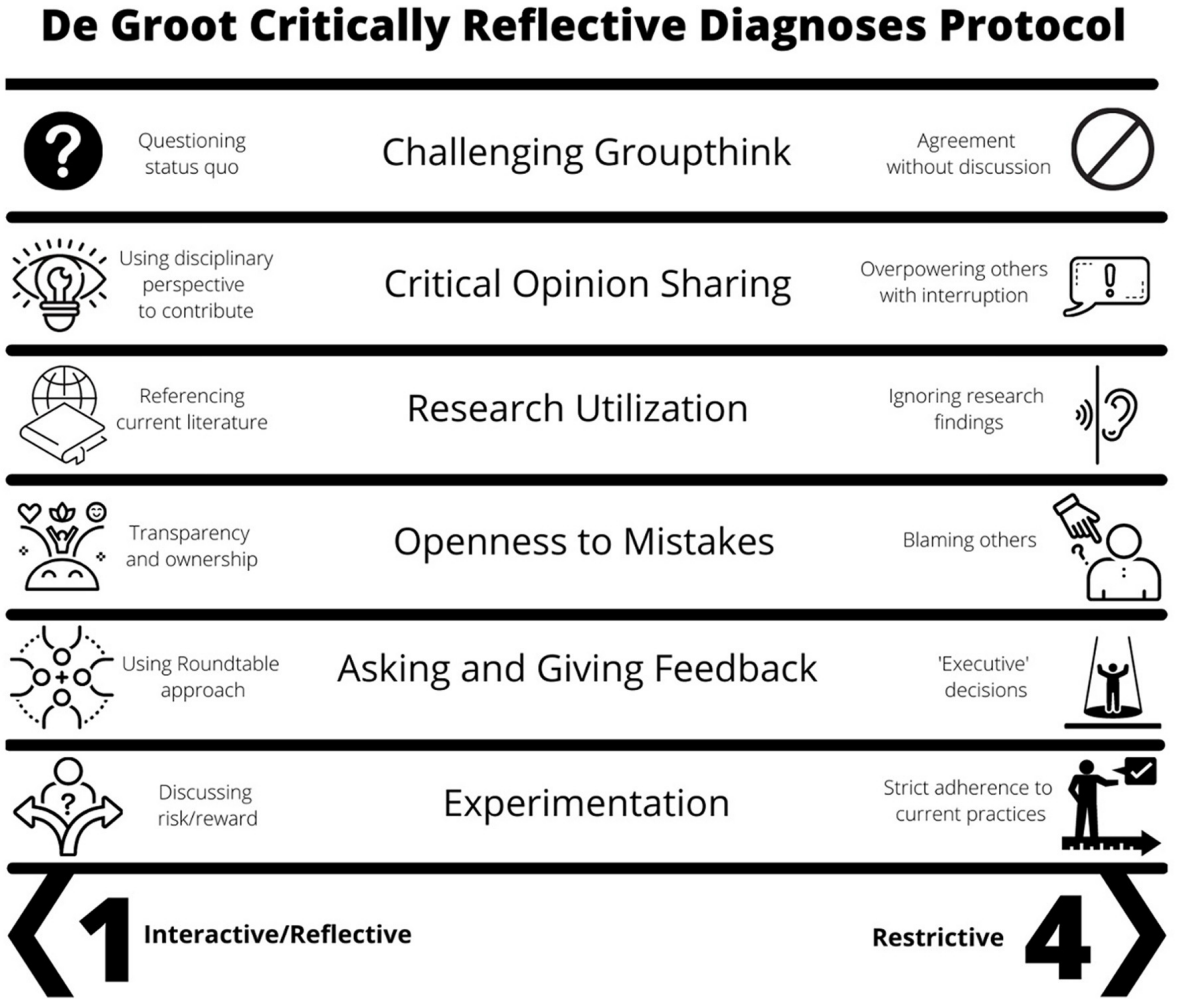
The de Groot Critically Reflective Diagnoses Protocol criteria. Scores from allocation meeting transcripts can range from 1 to 4 (best group dynamics to worst group dynamics).

Rather than relying on team member self-report of team dynamics impacting decision- making, the DCRDP provides 6 criteria for researchers to evaluate recorded team dialogue more objectively: challenging groupthink (embracing different opinions that differ from the dominant view), critical opinion sharing (sharing opinions that can be discussed openly), research utilization (discussing research), openness to mistakes (openly taking responsibility for errors), asking and giving feedback, and experimentation (thought experiment). The presence and strength of each aspect is supported by verbatim textual excerpts (codes) and given a numerical score ranging from 1 to 4. Teams with lower scores represent more interactive, reflective, and equitable group functioning while those with higher scores exhibit restrictive communication patterns, more dysfunctional group dynamics, and potentially more biased decision-making. To illustrate how the DCRDP is applied, see the Figure 1 for team decision-making patterns reflecting best and worst scores.

## Methods

This was a mixed-methods study that required the coordinated integration of both qualitative and quantitative data to uncover intricacies within complex healthcare phenomena (8). A qualitative descriptive approach was deemed appropriate for the qualitative portion of the study as a means of examining the elusive phenomenon of implicit bias using minimal abstraction (9, 10). In essence, in this study we sought to examine when, how, and in what forms bias appears during team-based clinical discussions using an established schematic in the form of the DCRDP, which provided a coding framework for analyzing dialogue patterns (6). While use of an implementation framework such as NPT is recommended to increase the effectiveness and sustainability of new approaches in healthcare, quantifying high-risk processes (such as multidisciplinary decision-making in allocation of heart transplant) using a tool such as the DCRDP may further explicate this complicated process in implementation science (7).

### Application of the de Groot Critically Reflective Diagnoses Protocol in a mixed-methods study

Although qualitative descriptive approaches are appropriate for evaluation of unguided group dialogue such as that occurring during therapy allocation meetings, analytic procedures in this method vary widely and may benefit from the use of additional tools to help codify and makes sense of the content (9, 10). Thus, the DCRDP aided this process by providing structure and increasing objectivity during qualitative data analysis through numerical quantification supported by textual codes. In this study, hierarchical logistic regression models were used which accounted for important individual factors (i.e., demographics and comorbidities) as well as accounting for DCRDP for a meeting and for variability among centers as well as among meetings within centers. The quantitative (scoring) portion of the DCRDP captured previously unexplored team dynamics and communication patterns in a numerical form, and these scores were added to regression models examining how group decision-making processes were associated with heart failure therapy allocation by race and gender.

### Data collection procedures

To explore these complex team dynamics, we audio recorded heart failure therapy allocation meetings at multiple healthcare centers across the United States (U.S.) and transmitted the recordings to Health Insurance Portability and Accountability Act (HIPAA) approved transcriptionists. Institutional Review Board approval was received from the University of Arizona. Verbal consent was obtained from team members participating in the allocation meetings prior to the series of recordings being collected. All identifiers were removed from the transcripts. For example, transcripts were blinded to heart allocation center sites and race/ethnicity/gender of all team members and patients under review for therapy allocation. Individual team members were differentiated in the transcripts numerically (i.e., Speaker 1, Speaker 2, etc.) and no other identifying characteristics were revealed.

### Data coding and analysis

Two data analysts with expert-level qualitative research experience separately coded the blinded transcripts in their entirety by using the DCRDP as a coding scheme. Exemplar phrases and excerpts illustrating repetitive ideas were categorized into each of the 6 criteria to support the given numerical score. Codes were selected both for frequency and for degree of alignment with each criterion. Consistent with a qualitative descriptive approach, the analysts performed content analysis using the DCRDP as a guide which controlled interpretation and facilitated the recognition of patterns based on the protocol (10). The Table 1 provides theoretical examples of exemplar quotes illustrating scores for each criterion.

**Table 1:** Codes illustrating scores for de Groot Critically Reflective Diagnoses Protocol criterion.

	**1 interactive and reflective**	**2 individual with reflection**	**3 not reflective nor interactive**	**4 restrictive**
Challenging groupthink	Before we make a final decision, what else should we consider about this patient?	As the transplant director, I feel strongly about this, but I would like to hear from the rest of the team before we move forward.	The surgical team will have the final say on this decision.	We think that this patient has too many unresolved social issues; we are not going to offer advanced therapies at this time.
Critical opinion sharing	The social work team are the best ones to answer that question. Can we hear from them first?	I have some concerns about this patient that I'd like to discuss with the group.	In my opinion, this therapy is rarely effective.	I'd like to interrupt and say that we've already heard this portion of the evaluation before.
Research utilization	We came across two newly published studies about this issue that we would like to present to the team.	I would like to consult the guidelines for insight about our next steps.	I don't know if there are any studies on this topic.	Regardless of what the research indicates, this patient is not a good candidate for transplant.
Openness to mistakes	Thank you for recognizing that the information in the patient's record is incorrect. I apologize for that and will correct it today.	The infectious disease team has been really overwhelmed lately, so I'll connect with them much earlier next time.	It's a complex situation and sometimes things get missed.	That team always drops the ball with our patients and it's very frustrating.
Asking and giving feedback	Does anyone have any additional insight into this patient?	I wasn't sure about initiating this medication, so I have some questions I'd like to ask the team.	If no one has any advice for how to improve this situation, let's move on with the discussion.	The patient's caregiver had some concerns, but we never spoke with them.
Experimentation	Would we be willing to try this therapy for the first time?	I'm not sure how that medication would impact the patient, but I'll look into it.	We should not be taking high risk patients like this at our center.	We have never tried that approach before, and I don't think we should now.

The examples provided for each code are theoretical examples. Exact quotes for the codebook have been previously published (14).

After each transcript was independently scored and exemplar supporting quotes were selected, the two analysts came together with the principal investigator to compare results. The researchers found significant congruence (>75%) between the independently scored transcripts for each of the 6 DCRDP criteria. In many cases, the same textual codes were also selected by both analysts to support the numerical score. This finding reflected a high level of intercoder reliability, a key aspect of qualitative research for ensuring transparency, consistency, reflexivity, and trustworthiness (11). When the analysts' numerical scores differed by 1 point the researchers selected the mean as the final score. When the two scores differed by more than 1 point, the two analysts critically reexamined the supporting codes with the principal investigator serving as an arbitrator during negotiations for the final score. Consensus was achieved on each of the numerical scores for each transcript with the two analysts selecting the most illustrative codes based on their deep familiarity with the data. Following completion of all coding, separate research team members unblinded each patient's race, ethnicity, and sex using patient data and order of discussion submitted by participating centers, which was used by the statistician to complete analyses. The association of DCRDP scores with allocation decisions according to patient race, ethnicity, or sex uncover a standardized method for identifying bias.

### Maintaining rigor

Study rigor was ensured through the following qualitative research procedures (9, 10). Credibility was promoted through researcher triangulation as the two qualitative analysts independently coded and scored each transcript with the principal investigator serving as an arbitrator when the numerical scores differed by more than 1 point. Having a minimum of two qualitative analysts separately code the data in its entirely followed by negotiated consensus with an arbitrator is a best practice in qualitative analysis to ensure reliability (11). Confirmability and reflexivity were achieved through regular debriefing between all three researchers during data analysis with notes documenting the decision-making processes and a clear audit trail located in an online data sharing platform (12). Reflexive notes and team debriefing was especially important considering that the two analysts and the principal investigator are all clinician-investigators with experience in team-based decision making; acknowledging these epistemological influences was essential during coding (12, 13). Transferability was encouraged through our demonstration of how the DCRDP can be used as a mixed-methods evaluation tool of team decision-making that can be adopted by others seeking to identify team functionality issues and design strategies to improve performance and reduce bias (10). Dependability was demonstrated by the easily traced verbatim quotes and their alignment with the codebook, which consisted of established DCRDP criteria.

While DCRDP scores and corresponding codes represent the etic, or outsider, viewpoint of allocation meetings, additional survey and interview analyses enacted in another phase of the study captured the emic, or insider, perspective of allocation team members as they engaged in group decision-making. Consideration of both perspectives strengthened the qualitative portion of this mixed-methods study and contributed to overall trustworthiness (8).

### DCRDP findings, strengths, and limitations

Analysis of meeting transcripts using the DCRDP combined with hierarchical logistic regression indicated that as team function scores improved, the probability of women being allocated advanced heart failure therapies increased and was statistically significant (*p* = 0.035) (14). Some centers exhibited consistently higher functioning team dynamics, although no statistically significant effect was observed for race and ethnicity (14).

We found that the use of a previously substantiated data analysis tool was both effective and efficient during deductive coding of team dialogue transcripts. The DCRDP provided a more objective measure of the frequency and strength of various communication patterns among advanced heart failure therapy allocation teams, as succinctly illustrated in the Table. In conjunction with additional analyses, the DCRDP proved to be a useful tool for examining how team communication patterns were related to treatment decisions for a diverse set of heart failure patients across several allocation centers in the U.S.

There were some limitations with using the DCRDP. Although codes provided evidence for the 6 DCRDP criteria, not all were represented in each transcript. As a result, the research team imputed the mean numerical value of missing criteria. This also meant that for some transcripts, there was a dearth of exemplar phrases or excerpts to illustrate certain criteria. For example, many transcripts lacked any evidence of the DCRDP criteria “research utilization.” While some allocation teams consistently failed to utilize research findings during their decision-making, we recognize that this aspect was potentially occurring outside of the recorded meetings in other cases. Another limitation was the inability to assess communication features such as body language or voice tone in the transcripts, both of which may factor into overall team dynamics and potentially biased decision-making. This limitation could be mitigated by including a research assistant acting as an observer during the meetings or through evaluation of video recordings of the meetings. However, either of these approaches would increase the risk for participant deidentification and could potentially lead to the Hawthorne effect influencing participant behavior during team meetings (15). A third limitation is that the DCRDP was originally developed with small professional groups of 5–7 people (7), and it is unclear how well DCRDP performs with larger number of active speakers. Although our application of the protocol was among larger meetings with over 20 speakers as is typical of transplant allocation teams, we demonstrated intercoder reliability using DCRDP.

## Discussion

The DCRDP is a compelling tool for evaluating bias in clinical group decision-making by addressing key aspects of team behavior and communication including challenging groupthink, critical opinion sharing, research utilization, openness to mistakes, asking and giving feedback, and experimentation. Through the quantification of these 6 major criteria as supported by textual excerpts, researchers can assess different aspects of team dynamics and functionality that may contribute to biased performance. The DCRDP may enhance the design and testing of implementation strategies underpinned by frameworks such as NPT. The general compatibility of NPT with additional tools (such as the DCRDP) is supported in the literature as a mechanism for widening our contextual understanding of human behavior (5).

While there is sufficient research addressing individual healthcare provider biases (1–3), the ability of the DCRDP to aid in the detection of team-based bias toward patients with marginalized racial and gender identities is promising and unique. Findings from the DCRDP could contribute to the design of group-level implementation strategies aimed at improving multidisciplinary communication and performance during collective decision-making. Post-intervention re-assessment or integration of the DCRDP into a surveillance program should be implemented since longitudinal measurements are essential for improving health equity among marginalized populations (4). As with our study, scores from the DCRDP can be incorporated into statistical models that include other data to comprehensively explore how clinical group functionality is associated with patient level outcomes.

In conclusion, we successfully applied the DCRDP to assess racial and gender bias among clinical teams responsible for allocating advanced heart failure therapies. Findings from this study contribute to the limited body of literature on potentially effective methods for assessing and implementing strategies to mitigate implicit bias among multidisciplinary clinical teams. Considering the persistence and insidious nature of patient inequities that are influenced by team-based decision-making, new methodological approaches in health and social science research are warranted to detect and mitigate group bias. The DCRDP has a wide application in implementation research by demonstrating a standardized method to evaluate group dynamics and bias.

## Data availability statement

The original contributions presented in the study are included in the article/supplementary material, further inquiries can be directed to the corresponding author.

## Ethics statement

The studies involving human participants were reviewed and approved by the Institutional Review Board from the University of Arizona. Written consent was obtained from all team members participating in the allocation meetings prior to the series of recordings being collected.

## Author contributions

Concept and supervision: KB. Manuscript draft: NP and MH. Critical review: All authors. All authors contributed to the article and approved the submitted version.
